# Survival in overweight patients with advanced pancreatic carcinoma: a multicentre cohort study

**DOI:** 10.1186/1471-2407-14-728

**Published:** 2014-09-29

**Authors:** Benjamin Kasenda, Annatina Bass, Dieter Koeberle, Bernhard Pestalozzi, Markus Borner, Richard Herrmann, Lorenz Jost, Andreas Lohri, Viviane Hess

**Affiliations:** Department of Medical Oncology, University Hospital Basel, Petersgraben 4, Basel, CH-4031 Switzerland; Basel Institute for Clinical Epidemiology and Biostatistics, University Hospital Basel, Basel, 4031 Switzerland; Department of Medical Oncology, Cantonal Hospital of St. Gallen, St. Gallen, 9007 Switzerland; Department of Medical Oncology, University Hospital of Zurich, Zurich, 8091 Switzerland; Department of Medical Oncology, University Hospital of Berne, Berne, 3010 Switzerland; Department of Medical Oncology, Cantonal Hospital Basel-Country, Basel-Country, 4101 Switzerland

**Keywords:** Pancreatic cancer, Body mass index, BMI, Prognostic factors, Survival, CA 19–9, Baseline

## Abstract

**Background:**

Obesity is a risk factor for developing pancreatic cancer. We investigated the impact of obesity on survival in patients diagnosed with locally advanced or metastatic pancreatic cancer.

**Methods:**

In a multicentre, retrospective study, we included all patients with advanced or metastatic pancreatic cancer treated at four Swiss hospitals between 1994 and 2004. We categorized patients into four body mass index (BMI) groups (<18.5, 18.5 – 25, ≥ 25 – 29, ≥30 kg/m^2^) and used multivariable Cox regression to investigate the impact of BMI on survival. Missing data were handled using multiple imputations.

**Results:**

483 patients were included. Median age was 66 years (range 59–74), 47% were female, 82% had stage IV disease, 72% had an ECOG below 2, and 84% were treated with gemcitabine-based first-line chemotherapy. After a median follow-up of 8.5 months, 6 and 12-month survival probabilities of the whole cohort were 67% (95% CI 63% - 71%) and 37% (95% CI 33% - 42%), respectively. Unadjusted 12-month survival rates in each BMI group were: 48% (95% CI 33% - 62%), 42% (95% CI 36% - 48%), 30% (95% CI 22% - 38%), and 11% (95% CI 4% - 24%), respectively. In multivariable analysis, increasing BMI (HR 1.22, 95% CI 1.04 – 1.41, p = 0.012) and CA 19–9 (HR 1.07, 95% CI 1.02 – 1.11, p = 0.003) were significantly associated with worse survival prognosis. Patients with a good clinical performance status (ECOG < 2) had a better prognosis (HR 0.76, 95% CI 0.65 – 0.96, p = 0.019).

**Conclusions:**

Obese patients diagnosed with advanced pancreatic cancers have a worse prognosis compared to non-obese patients. BMI should be considered for risk stratification in future clinical trials.

**Electronic supplementary material:**

The online version of this article (doi:10.1186/1471-2407-14-728) contains supplementary material, which is available to authorized users.

## Background

Advanced pancreatic adenocarcinoma is the fifth leading cause of cancer-related deaths and carries a devastating prognosis with 5-year survival rates of 6% [1, 2]. Incidence increases with age and most cases are diagnosed above the age of 50 years at an unresectable stage of disease [3]. Obesity, smoking and dietary factors such as red meat consumption are risk factors for developing pancreatic cancer [4, 5]. Moreover, physical activity seems to decrease the risk of developing pancreatic cancer, especially among those who are overweight [6]. Underlying mechanisms behind these associations are not fully elucidated yet, but hyperinsulinaemia and insulin resistance are considered key factors [7]. For patients with established diagnosis of advanced pancreatic cancer only few prognostic factors have been described. The most consistent one is a reduced clinical performance status at the time of diagnosis, which is associated with decreased survival [8]. Recently high baseline serum concentration of the tumour marker CA 19–9 has been identified as an independent negative prognostic factor in two independent cohorts of patients with advanced pancreatic cancer [8, 9]. There is some evidence that men have an increased risk of death compared to women [9], however this association was not confirmed by another group [10]. Obesity at the time of diagnosis has been reported to be associated with worse prognosis in several malignant diseases including prostate, colon, and breast cancer [11–13] – an observation with major implications given the epidemic prevalence of obesity in many regions of the world. In the present study we investigated the impact of body mass index (BMI) on survival in patients with advanced pancreatic carcinoma accounting for known prognostic factors such as baseline CA 19–9 concentration and clinical performance status.

## Methods

### Eligibility criteria and study design

All patients with stage III/IV pancreatic carcinoma treated at four Swiss hospitals between 1994 and 2004 were included, irrespective of age, PS, histopathological grade or treatment modality. Patient data were retrospectively collected using a pre-specified case report form that recorded anonymized data about patient and tumour characteristics at baseline, treatment, and follow-up. All identified eligible patients from the participating centres were included. Case report forms were checked for consistency and queries re-checked with each centre before entering the data in a central database. The research ethics committee of Basel (EKBB) approved this study.

### Statistical considerations

We calculated overall survival (OS) from the time of diagnosis until death due to any cause and estimated the OS probabilities using the Kaplan-Meier method. Follow-up was calculated for all patients irrespective of survival status. We categorized BMI (body weight in kilograms/[height in metres]^2^) according to four groups (<18.5, 18.5 – 25, ≥ 25 – 29, and ≥ 30) as proposed by the World Health Organization [14]. For illustration, we plotted corresponding Kaplan Meier curves of each BMI group. These unadjusted survival probabilities were compared using the log-rank test; we also calculated unadjusted hazard ratios (HRs) with 95% confidence intervals for these categories with BMI 18.5 – 25 as the reference group. In an explorative unadjusted survival analysis, we also investigated the association between insulin and metformin intake on OS, respectively.

In our primary prognostic analysis, we investigated the impact of BMI at time of diagnosis on OS using multivariable Cox regression analysis adjusted for the following baseline characteristics: CA 19–9 serum concentration (log transformed because of a very skewed distribution), age (as continuous variable), sex, presence of diabetes, performance status (Eastern Cooperative Oncology Group [ECOG] <2 *versus* ECOG ≥ 2), and stage of disease (III *versus* IV). In addition, we conducted one sensitivity analysis to investigate the robustness of the prognostic impact of BMI. In this analysis, we only considered those patients who received gemcitabine-based first-line therapy. Because BMI is an inherently continuous variable, in all regression analyses, we included BMI as a continuous variable as recommended [15] to circumvent the well-known analytical pitfalls including loss of power and spurious findings by arbitrarily choosing cut-offs or categories [16]. For all survival analyses, missing data were imputed using multiple imputations using chained equations [17, 18]. In addition, we conducted bootstrap procedures for internal model validation [19]. We present HR and 95% confidence intervals from univariable analysis of each risk factor, but irrespective of significance, they were included in the multivariable analyses. The assumptions of proportional hazards were investigated graphically and tested using the Grambsch-Therneau test. P-value of < 0.05 (two-sided) was considered significant. We used the statistical program STATA version 13.1 (STATA Corp, Texas, USA) for analysis (see Additional file 1 for further details about the statistical analyses).

## Results

### Patient characteristics

All 483 patients with advanced pancreatic cancer identified between 1994 and 2004 were included in our analyses; characteristics are summarized in Table 1 stratified by BMI group. The majority was diagnosed with stage IV disease; 41 patients (8.5%) were treated with best supportive care only. The vast majority of patients underwent first-line treatment with gemcitabine single agent (*N* = 286; 59%) or in combination with capecitabine (*N* = 65; 14%), or cisplatin (*N* = 22; 5%) (Table 1). 186 (39%) patients received a second line therapy after progression; 3rd, 4th, and 5th line therapy was applied in 51 (11%), 13 (3%), and 3 (1%) patients, respectively.

**Table 1:** **Baseline characteristics of all included 483 patients with advanced pancreatic cancer**

Characteristics	BMI < 18.5	BMI 18.5 - 25	BMI 25 - 30	BMI > =30	Total
	N = 44	N = 294	N = 113	N = 32	N = 483
Age at diagnosis					
Median(IQR)	64.3 (55.4, 73.6)	67.1 (58.9, 74.2)	66.6 (58.8, 74.1)	63.7 (58.2, 67.2)	66.4 (58.6, 74)
Sex					
Female	24 (54.5)	135 (45.9)	36 (31.9)	13 (40.6)	208 (43.1)
Male	20 (45.5)	159 (54.1)	77 (68.1)	19 (59.4)	275 (56.9)
ECOG at diagnosis					
ECOG > =2	9 (20.5)	64 (21.8)	27 (23.9)	5 (15.6)	105 (21.7)
ECOG < 2	35 (79.5)	230 (78.2)	86 (76.1)	27 (84.4)	378 (78.3)
Stage at diagnosis					
III	8 (18.2)	58 (19.7)	22 (19.5)	3 (9.4)	91 (18.8)
IV	36 (81.8)	236 (80.3)	91 (80.5)	29 (90.6)	392 (81.2)
CA19-9 at diagnosis					
Median (IQR)	392.1 (53.2, 2589.8)	546.1 (66.5, 3644)	801 (134.7, 4390)	689 (207.7, 14630.7)	609.6 (84.4, 3819.8)
Diabetes at diagnosis					
Diabetes	10 (22.7)	74 (25.2)	29 (25.7)	14 (43.8)	127 (26.3)
No-diabetes	34 (77.3)	220 (74.8)	84 (74.3)	18 (56.2)	356 (73.7)
RTX at any therapy line					
No-RTX	43 (97.7)	273 (92.9)	108 (95.6)	30 (93.8)	454 (94)
RTX	1 (2.3)	21 (7.1)	5 (4.4)	2 (6.2)	29 (6)
Most frequent 1st line chemotherapies					
Gem	28 (63.6)	173 (58.5)	68 (60.2)	17 (53.1)	286 (59.2)
Gem + Cap	6 (13.6)	39 (13.3)	14 (12.4)	6 (18.8)	65 (13.5)
Gem + Cis	1 (2.3)	14 (4.8)	7 (6.2)	0 (0)	22 (4.6)
BSC	6 (13.6)	23 (7.8)	10 (8.8)	2 (6.2)	41 (8.5)

All values are numbers (percentages) unless otherwise specified. Median BMI was 23 (IQR 21–25). *Abbreviations*: *BMI* body mass index in kg / [height in m]^2^, *BSC* best supportive care, *Cap* capecitabine, *Cis* cisplatin, *ECOG PS* Eastern Cooperative Group Performance Status, *Gem* gemcitabine, *IQR* inter quartile range, *RTX* radiotherapy.

### Overall survival

**Figure 1:**
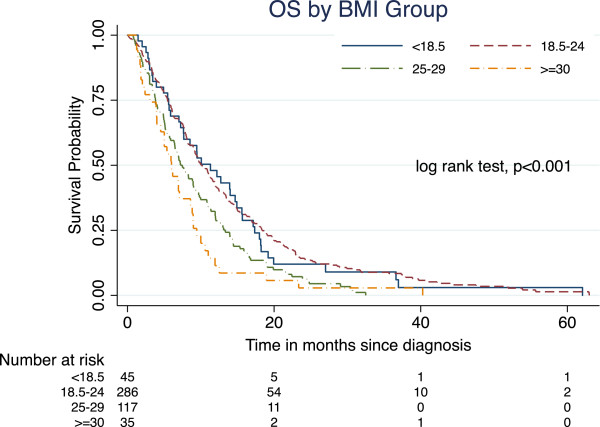
**Overall survival by BMI group.** Abbreviations: BMI = body mass index; OS = overall survival.

After a median follow-up of 8.5 months (interquartile range 2 – 15 months), 448 patients (93%) had deceased. The respective 6, 12, and 24-month survival probabilities for the whole cohort were 67% (95% CI, 63% - 71%), 37% (95% CI, 33% - 42%), and 11% (95% CI, 8% - 14%). Figure 1 illustrates the OS probabilities grouped by the four BMI groups following the WHO classification. The unadjusted 12-month survival rates in each BMI group were: 48% (95% CI 33% - 62%), 42% (95% CI 36% - 48%), 30% (95% CI 22% - 38%), and 11% (95% CI 4% - 24%), respectively. The unadjusted HR for each BMI group was: 1.06 (95% CI 0.76 - 1.46, p-value = 0.748, BMI < 18.5), HR 1.00 (reference, BMI 18.5 – 25), 1.53 (95% CI 1.22 - 1.91, p-value < 0.001, BMI ≥ 25 – 29), and 2.02 (95% CI 1.42 - 2.89, p-value < 0.001, BMI ≥ 30).

### Primary prognostic analyses

Increasing BMI adversely affected survival; each increase in 5 units was independently associated with 21% relative risk increase of death in our primary multivariable analysis (HR 1.21, 95% CI 1.06 – 1.41, p-value = 0.004). Patients with a good clinical performance status (ECOG < 2) had a smaller risk of death compared to patients with a worse performance status (HR 0.76, 95% CI 0.65 – 0.96, p-value = 0.019). Increased CA 19–9 (on log scale) was significantly associated with decreased survival (1.07, 95% CI 1.02 – 1.11, p-value = 0.003). Disease stage had no impact on survival prognosis (Table 2). All findings were confirmed in the internal model validation using bootstrap procedures. In 127 patients with known and treated diabetes at baseline, insulin (treatment in 49/127 (38.6%) cases) or metformin (treatment in 10/127 (7.9%) cases) had no impact on OS, respectively (insulin, log-rank test *p =* 0.9687; metformin, log-rank test *p* = 0.2023).

**Table 2:** **Primary prognostic analysis of mortality using multivariable Cox regression (N = 483)**

Variable	Univariable			Multivariable		
	HR	95% CI	***P*** -value	HR	95% CI	***P*** -value
BMI	1.25	1.10 - 1.42	<0.001	1.21	1.06 - 1.41	0.004
(increments of 5 units)						
Age (continuously)	1.00	0.99 - 1.00	0.865	1.00	0.99 - 1.01	0.920
Performance status	0.77	0.62 -0.97	0.024	0.76	0.65 - 0.96	0.019
(ECOG < 2 *versus* ECOG > =2)						
Diabetes	0.94	0.76 -1.16	0.540	0.92	0.74 -1.14	0.438
(yes *versus* no)						
Stage	0.84	0.65 -1.08	0.188	0.92	0.70 - 1.20	0.525
(stage III *versus* stage IV)						
Sex	1.23	1.02 - 1.48	0.033	1.16	0.95 - 1.41	0.145
(male *versus* female)						
CA 19-9	1.08	1.04 -1.13	<0.001	1.07	1.02 - 1.11	0.003
(continuously on log scale)						

*Abbreviations*: *BMI* body mass index, *CI* confidence interval, *ECOG* Eastern Cooperative Oncology Group, *HR* hazard ratio.

### Sensitivity analyses

In the subpopulation of patients who received gemcitabine-based therapy (*N* = 407, Table 3) the prognostic impact of BMI was little more pronounced with a 28% increase of risk of death for each BMI increase by 5 units (HR 1.31, 95% CI 1.10 - 1.50, p < 0.001). Disease stage also had no impact on survival prognosis in this sensitivity analysis. All results were confirmed by analyses based on the bootstrap sample. In addition, male patients showed a relative risk increase of 30% compared to women, however, this association was not consistent with our primary analysis.

**Table 3:** **Sensitivity analysis of mortality using multivariable Cox regression techniques**

	Univariable			Multivariable		
Variable	HR	95% CI	P-value	HR	95% CI	P-value
BMI	1.30	1.13 - 1.48	<0.001	1.28	1.10. – 1.50	0.001
(in steps of 5)						
Age (continuously)	1.00	0.99 - 1.01	0.695	1.00	0.99 - 1.01	0.405
Performance status	0.73	0.60 - 0.94	0.015	0.70	0.54 - 0.91	0.009
(ECOG < 2 versus ECOG ≥ 2)						
Diabetes	0.87	0.69 - 1.09	0.234	0.78	0.61 - 1.01	0.064
(yes versus no)						
Stage	0.98	0.74 - 1.30	0.912	1.10	0.80 - 1.47	0.601
(stage III versus stage IV)						
Sex	1.35	1.10 - 1.66	0.004	1.30	1.04 -1.63	0.023
(male versus female)						
CA 19-9	1.08	1.04 - 1.12	<0.001	1.06	1.02 - 1.11	0.006
(continuously on log scale)						

Only patients treated with gemcitabine-based first-line therapy were included (N = 407). *Abbreviations*: *BMI* body mass index, *CI* confidence interval, *ECOG* Eastern Cooperative Oncology Group, *HR* hazard ratio.

## Discussion

### Summary of findings

Overweight is associated with shortened survival in a cohort of European patients with advanced pancreatic cancer, independently of known prognostic factors including high CA 19–9 serum concentration at baseline, clinical performance status and disease stage (III versus IV).

### Strengths and limitations

Our data are based on a representative sample of patients diagnosed and treated at four oncological centres in Switzerland – one of the first cohorts of *European* patients describing an association between BMI and prognosis. Furthermore, our dataset allowed adjusting for baseline serum CA 19–9 concentration, a recently recognized important prognostic factor [8, 9], clinical performance status, and stage of disease. In addition, we report detailed information on treatment regimens and investigated the independent prognostic role of increased BMI also in the more homogenous patient group treated with gemcitabine-based first-line therapy – a valid standard treatment option, especially for elderly or frail patients [20]. Our results proved robust in sensitivity analyses using bootstrap sampling [19], a state of the art internal validation technique, which further strengthens the evidence for BMI being an independent prognostic factor.

Because of the retrospective design, several limitations have to be addressed. First, in 29% BMI data were missing. To circumvent the loss of power, risk of bias, and to use all information available in the dataset for our analyses, we used multiple imputations to impute missing values. This approach has been proposed as a remedy for such situations and its incorporation into routine practice has been recommended to avoid biased estimates [17, 21, 22]. Also, the absolute number of obese and very obese patients (BMI >30) in our Swiss cohort was much lower (N = 32; 7%, Table 1) as compared to previously published US cohorts [10]. Second, we had no data on the development of BMI or onset of diabetes during therapy and follow-up. Therefore we could not investigate whether gain or loss of BMI would be of prognostic relevance over time. This could be of interest, because loss of appetite and weight are common problems during treatment of patients suffering from advanced/metastatic pancreatic cancer. Third, BMI as an indicator itself has some inherent limitations: e.g. elderly patients tend to have a shift of fat from peripheral to central sites with a concomitant increase in waist-to-hip ratio [23] which is not reflected in BMI. For such populations, and with evidence of health risks associated with abdominal (visceral) fat, the waist-to-hip ratio and waist circumference, have been commonly used in epidemiological studies [23], but were not available in our dataset.

### Compared to other studies

Our findings are in line with previous studies in cohorts of US adults, which included a higher percentage of obese (BMI ≥ 30) and very obese (BMI ≥ 35) patients and relied on self-reported BMI data (weight and height) as opposed to our measured data. In a Mayo Clinic cohort of patients with all stages of pancreatic cancer, including patients with early stage-disease who underwent surgery, Mc Williams et al. report that BMI at diagnosis has a negative impact on survival [10]. This was particularly pronounced in the very obese patients with a BMI of 35 to 39.99 kg/m^2^ (HR 1.32, 95% CI 1.08-1.62) and > 40 kg/m^2^ (HR 1.60, 95% CI 1.26-2.04), respectively. However, this analysis did not consider clinical performance status or CA 19–9 at baseline. In a case–control study designed to assess the risk of developing pancreatic cancer in overweight persons, Li et al. describe a shorter survival for patients who were overweight or obese during the year prior to diagnosis [4]. Based on 609 patients with all stages of pancreatic cancer included in the survival analyses, the association between obesity and overall survival was stronger among patients with resected tumours (HR 3.35, 95% CI, 1.50 - 7.49) than among those with unresected tumours (HR, 1.64, 95% CI 1.15 - 2.33). In patients with metastatic disease, obese patients (BMI ≥ 30) were at higher risk of death compared to normal weight patients (HR 1.57, 95% CI 1.03 - 2.40) [4]. Interestingly in a recent report from the nurses’ health study and the health professionals follow-up study [24], higher *pre*-diagnosis BMI – as far as 20 years prior to diagnosis – was also found to be associated with a shorter time from diagnosis to death.

In our univariable prognostic analysis, there was a trend towards better prognosis for patients with stage III disease as compared to patients with stage IV disease (HR 0.84, 95% CI 0.65 - 1.08), however this association did not reach the pre-defined level of statistical significance. Furthermore, in our primary *multivariable* analysis, this positive effect was not confirmed at all (HR 0.92, 95% CI 0.70 - 1.20), suggesting that BMI, baseline CA 19–9, and ECOG performance status are the most important prognostic factors in advanced and metastatic pancreatic cancer.

### Explanations for the association

Mechanisms behind the association between elevated BMI and decreased survival are still unknown. Pancreatic cancer is a very rapidly progressing fatal malignancy, thus most patients die because of uncontrollable disease. Deaths due to comorbidities associated with obesity, such as cardiovascular disease, are very unlikely to explain the reduced overall survival. Chronic hyperinsulinaemia and accompanying increased circulating C-peptide concentrations have been suggested as a potential link between obesity and the *development* of cancer in preclinical [25] and epidemiological studies [26, 27]. Consequently, we hypothesize that the metabolic environment, in which the cancer arises, might shape its biological behaviour and therefore response to treatment and prognosis. However, diabetes itself or diabetes treatment (insulin or metformin) was not of prognostic value in our cohort.

On a practical level, under-dosing of chemotherapy in obese patients may lead to decreased survival. Also, patients who were very obese received less second-line chemotherapy in our cohort: Only 6 out of 17 (24%) very obese patients (BMI ≥ 30) received at least one salvage therapy after first-line palliative therapy which was less compared to patients from lower BMI groups (<18.5, 10/31 [32%]; 18.5 – 25, 69/211 [33%]; ≥ 25 – 29, 27/75 [36%]). One could assume that patients with very low BMI (<18.5) would also have a worse survival prognosis, because very low BMI may indicate higher frailty and malnutrition at diagnosis. In fact, epidemiological studies on healthy Caucasian individuals show that the association between BMI and all cause mortality is best described by a J-shaped curve [28]. However, similar to another US study [10], in our analysis there was no difference between patients from the lowest BMI group (BMI < 18.5) compared to normal weight patients (BMI 18.5 – 25.0). Given the relatively small sample size (BMI < 18.5, N = 44), one can still assume that this possible association just did not become evident because of limited statistical power. In addition, around 80% of patient with BMI < 18.5 had a good clinical performance status and we cannot rule out that patients with worse performance status never presented to one of the participating centers.

### Implications for clinical practice and further research

Results from our study and others suggest that metabolic factors associated with obesity play a central role in the development and progression of pancreatic cancer. An immediate implication for clinical researchers is to include obesity (e.g. BMI ≥30) as a stratification variable in randomized clinical trials for patients with pancreatic cancer. Furthermore, drug dosing and distribution in the rapidly growing population of obese and very obese patients deserves further systematic investigations. Molecular characterization of malignant disease in the chronically overweight will reveal whether cancer that has arisen in the metabolic environment of obese patients is indeed a different disease. Finally, further research is needed to assess whether changes in body weight, e.g. through exercise interventions, with increase in lean body mass and/or decrease in adipose tissue alters not only some quality of life items [29] but also response to treatment and prognosis.

## Conclusion

Overweight patients with advanced or metastatic pancreatic carcinoma have a shortened survival compared to normal weight patients. Mechanisms explaining the association between increased BMI and worse prognosis are still not fully understood. However, because BMI is such a simple clinical marker, its strong prognostic value in patients with pancreatic cancer should be considered for risk stratification in future trials.

## Electronic supplementary material

Additional file 1: **Multiple imputations and bootstrap procedure.** (DOCX 21 KB)
