# Supplementary material for: Survival in overweight patients with advanced pancreatic carcinoma: a multicentre cohort study
Source: BMC Cancer. 2014 Sep 29;14:728. doi: 10.1186/1471-2407-14-728 (PMC4242603; doi:10.1186/1471-2407-14-728)
Supplement: Supplementary file 1 — Additional file 1: Multiple imputations and bootstrap procedure. (DOCX 21 KB) [file 12885_2014_5022_MOESM1_ESM.docx]

**Additional file 1**

**Multiple imputations**

We used the Gaussian normal regression multiple imputation method to impute missing values of all variables considered in our Cox regression models. We assumed that data were missing at random. The following variables were imputed: CA 19-9 (on log scale, because it had a severely skewed distribution), patient height, patient weight, and clinical performance status. The Nelson-Aalen estimator was included into the imputation equations. In total, 50 imputation sets were generated after a burning in of 10 iterations. Body mass index was calculated based on the imputed values of height and weight.

**Bootstrap procedure**

For internal model validations, we arbitrarily chose the 1^st^, 5^th^ 10^th^, and 15^th^ imputed data set separately to conduct the non-parametric bootstrap estimation procedure with 700 replications to investigate the robustness of our regression estimates.
